# Merkel cell carcinoma overlapping Bowen’s disease: two cases report and literature review

**DOI:** 10.1007/s00432-024-05743-0

**Published:** 2024-04-26

**Authors:** Xueqin Chen, Xiao Song, Hui Huang, Lian Zhang, Zhiqiang Song, Xichuan Yang, Shanchuan Lei, Zhifang Zhai

**Affiliations:** 1 Department of Dermatology, Southwest Hospital, Army Medical University, No. 30, Gaotanyan Street, Shapingba District, Chongqing, 400038 China; 2 Department of Dermatology, Yongchuan Hospital of Chongqing Medical University, No. 439, Xuanhua Road, Yongchuan District, Chongqing, 402160 China

**Keywords:** Merkel cell carcinoma, Bowen’s disease, Squamous cell carcinoma in situ, Histopathology, Therapy

## Abstract

**Purpose:**

Merkel cell carcinoma (MCC) is a rare neuroendocrine tumor of the skin, which mainly occurs in the sun exposed sites of white patients over 65 years, with a higher recurrence and metastasis rate. Clinically, MCC overlapping Bowen’s disease (BD) is a very rare subtype of MCC. Few cases in the literature have been described and the management is not well defined. We summarize and update the epidemiology, clinical and histopathological features, metastasis characteristics, local recurrence rate and management of it by presenting two cases of MCC overlapping BD and reviewing the literature over the last 11 years.

**Design:**

We consulted databases from PubMed, ResearchGate and Google Scholar by MeSh “Merkel cell carcinoma” and “Bowen’s disease”, “Bowen disease” or “squamous cell carcinoma in situ”, from January 2013 to December 2023 and reviewed the literatures. We reported two additional cases.

**Results:**

Total 13 cases of MCC overlapping BD were retrospectively analyzed, in whom mainly in elderly women over 70 years, the skin lesions were primarily located on the faces, followed by the extremities and trunk. Most of them were asymptomatic, firm, dark red nodules arising on rapidly growing red or dark brown patches, or presenting as isolated nodules. Dermoscopy evaluation was rarely performed in the pre-operative diagnostic setting. All cases were confirmed by histopathology and immunohistochemistry. The most definitive treatment was extended local excision, but local recurrences were common. Of the 13 cases, 4 cases experienced local or distant metastasis. One suffered from an in-transit recurrence of MCC on the ipsilateral leg after local excision and lymph node dissection, whose metastasis completely subsided after avelumab treatment and without recurrence or metastasis during 6 months of follow-up.

**Conclusions:**

MCC overlapping BD is a very rare skin tumor mainly predisposed on the faces, with high misdiagnosis rate and recurrence rate. Advanced disease at diagnosis is a poor prognostic factor, suggesting that earlier detection may improve outcome. The acronym, AEIOUN, has been proposed to aid in clinical identification. Our reports and the literature review can provide a better awareness and management of it.

## Introduction

Merkel cell carcinoma (MCC) is a rare and highly aggressive primary cutaneous neuroendocrine carcinoma, which predominantly affects individuals of Caucasian descent. Risk factors include advanced age, exposure to ultraviolet radiation, male gender, immunosuppression, hematologic malignancies or posttransplant status, and infection with Merkel cell polyomavirus (MCPyV) (Harms et al. 2018). It is characterized by high invasiveness, frequent local recurrence, a tendency for regional lymph node and distant metastases, with high mortality rates of 33–46% (Harms 2017; Garcia-Carbonero et al. 2019).

MCC often occurs in sunexposed sites, typically presenting as solitary nodules or patches with skin-colored, red or purple hues. Reportedly, it occured concomitantly with or in the setting of pre-existing cutaneous neoplasms, including actinic keratosis, Bowen’s disease (BD), squamous cell carcinoma (SCC), basal cell carcinoma (BCC) and miscellaneous adnexal tumors (Kervarrec et al. 2022). Only a small percentage of MCC presented combined with other tumors, for which, current data have suggested a more aggressive course than pure MCC (Tono et al. 2015; Chattopadhyay et al. 2020). Clinically, its association with BD is exceedingly uncommon. Its unspecific manifestations often lead to delayed diagnosis clinically, which is necessary for dermatologists and oncologists to familiarize themselves with and recognize it (Swain et al. 2022).

Few cases in the literature have been described and the management is not well defined. In our paper, we reviewed the literature and reported two additional cases to summarize the epidemiology, clinical and histopathological characteristics and management of it.

## Design

We first reported two cases with MCC overlapping BD. Then, we searched different databases, including PubMed, ResearchGate and Google Scholar by the combination MeSh of “Merkel cell carcinoma” and “Bowen’s disease, “Bowen disease” or “squamous cell carcinoma in situ” from January 2013 to December 2023. Total 15 papers were identified. Inclusion criteria were systematic review or meta-analysis of randomized controlled trials, review, retrospective comparative reviews/studies and case series. Exclusion criteria were laboratory studies and non-English translated articles. A wide review of the bibliography of each of the selected articles was performed. In total, 10 papers met our inclusion criteria, including 11 case reports and case series.

We reviewed and analyzed all of the cases with MCC overlapping BD and summarized the demographic information, such as the age, sex and the medical history, clinical and histopathological characteristics and the treatment and prognosis of them.

## Results

### Case reports

**Fig. 1:**
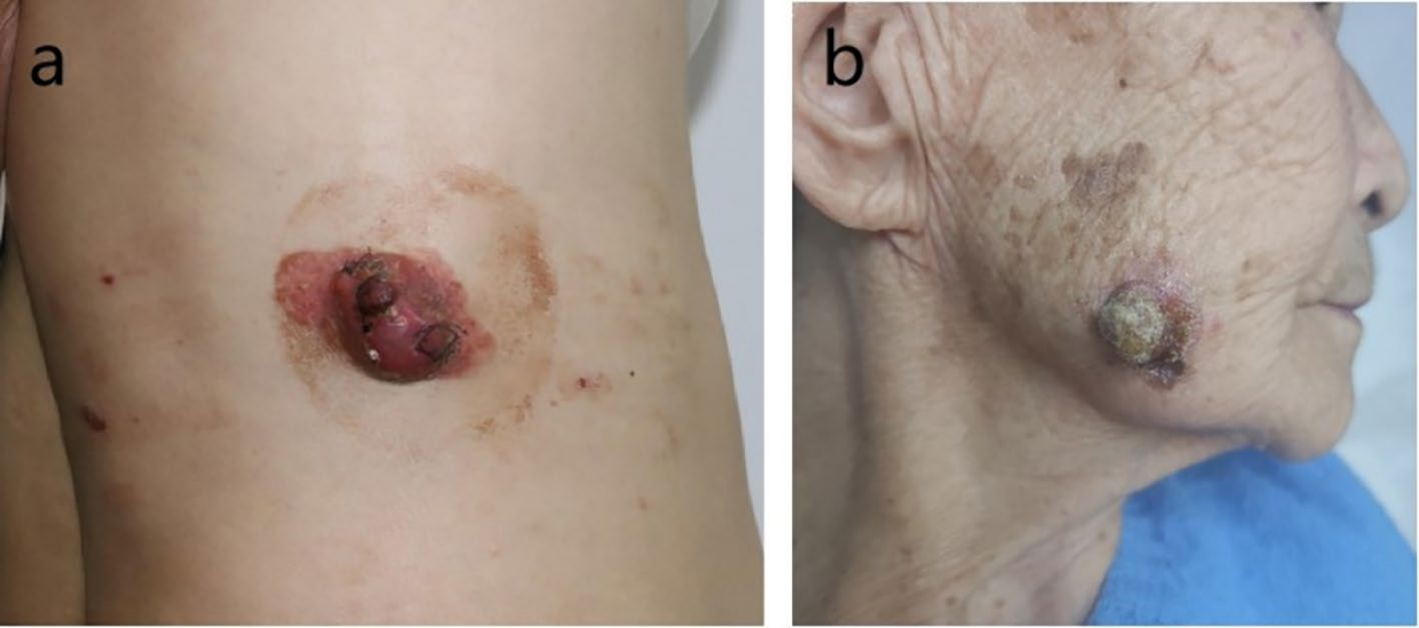
Clinical aspect of an MCC overlapping BD. **a** Solitary and dome shaped reddish nodule surrounded by an erythematous scaly patch on the right waist. **b** Ovoid dark erythematous painless tumor mass on the right mandibular angle, with peeling and scabbing on the surface of the mass

**Case 1** A 51-year-old man presented with a pruritic erythema on the right waist for over 5 years. In the past years, he paid no attention to it, though a dull red nodule had developed and gradually enlarged on the erythema. Physical examination revealed a 5 cm × 3 cm oval-invasive erythema covering with some scale, in the center of which a dull red, solid, non-tendor, well-demarcated nodule measuring 3 cm × 2 cm × 2 cm protruded from the skin surface. Significant hyperplasia of dilated capillaries, and a few scales can be seen on the surface of the neoplasm (Fig. 1a). Thoracoabdominal CT revealed no significant abnormalities, and peripheral blood count and tumor marker tests were normal.

**Case 2** An 87-year-old female presented with an asymptomatic neoplasm on the right maindibular angle for over a year. Physical examination showed a dull red patch measuring 2 cm × 1.5 cm , with a solid, well-defined and protruding nodule in the center measuring  1.5 cm × 1.5 cm × 1 cm, and with some scaling (Fig. 1b). There were no positive findings by the cranial and thoracoabdominal CT scans.

**Fig. 2:**
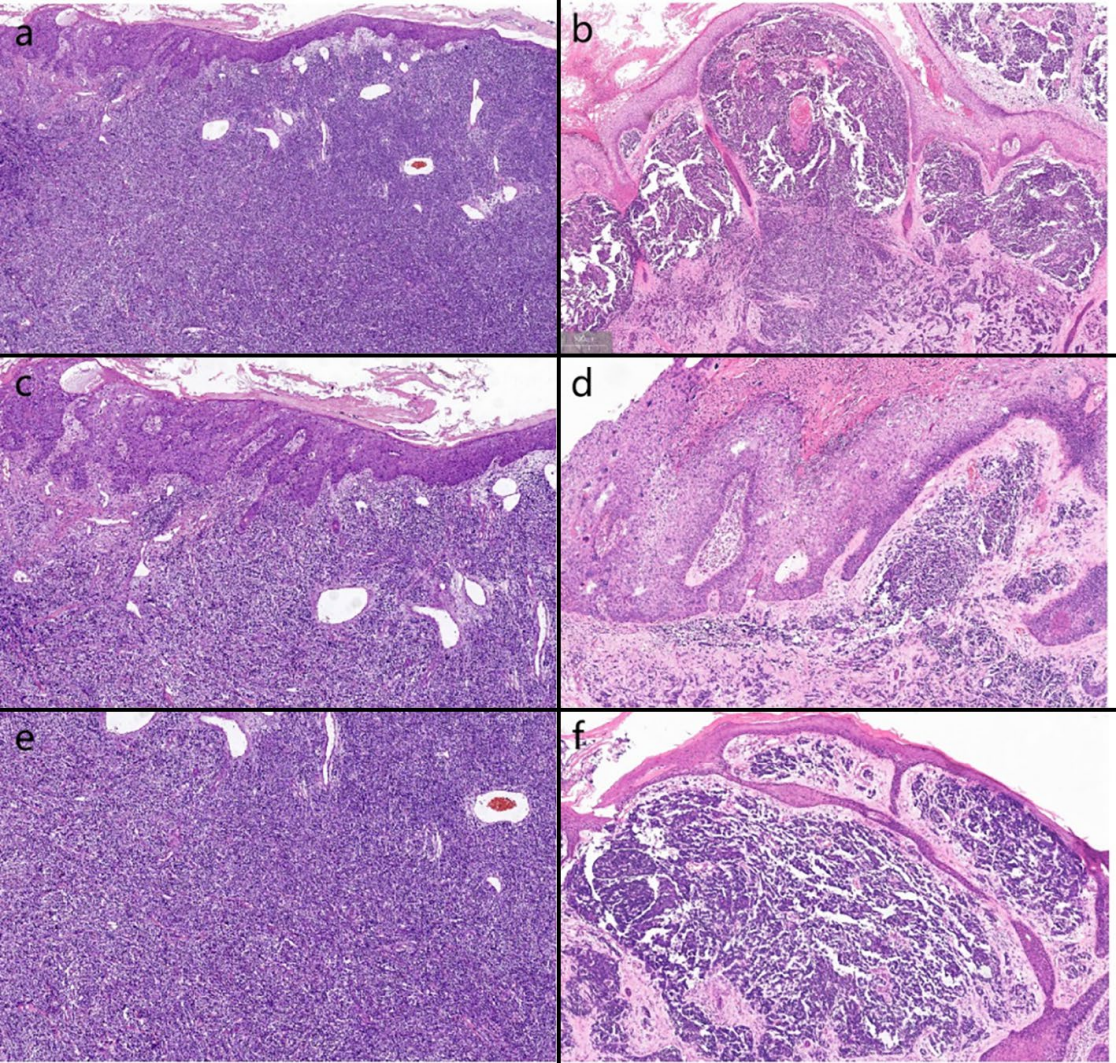
**a, b** On histopathology, BD is juxtaposed or strictly intermingled with MCC (H&E, ×4). **c, d** BD shows full thickness of atypical squamous cells (H&E, ×10). **e, f** Dermal dense infiltration of small round hyperchromatic small cells (H&E, ×10)

**Fig. 3:**
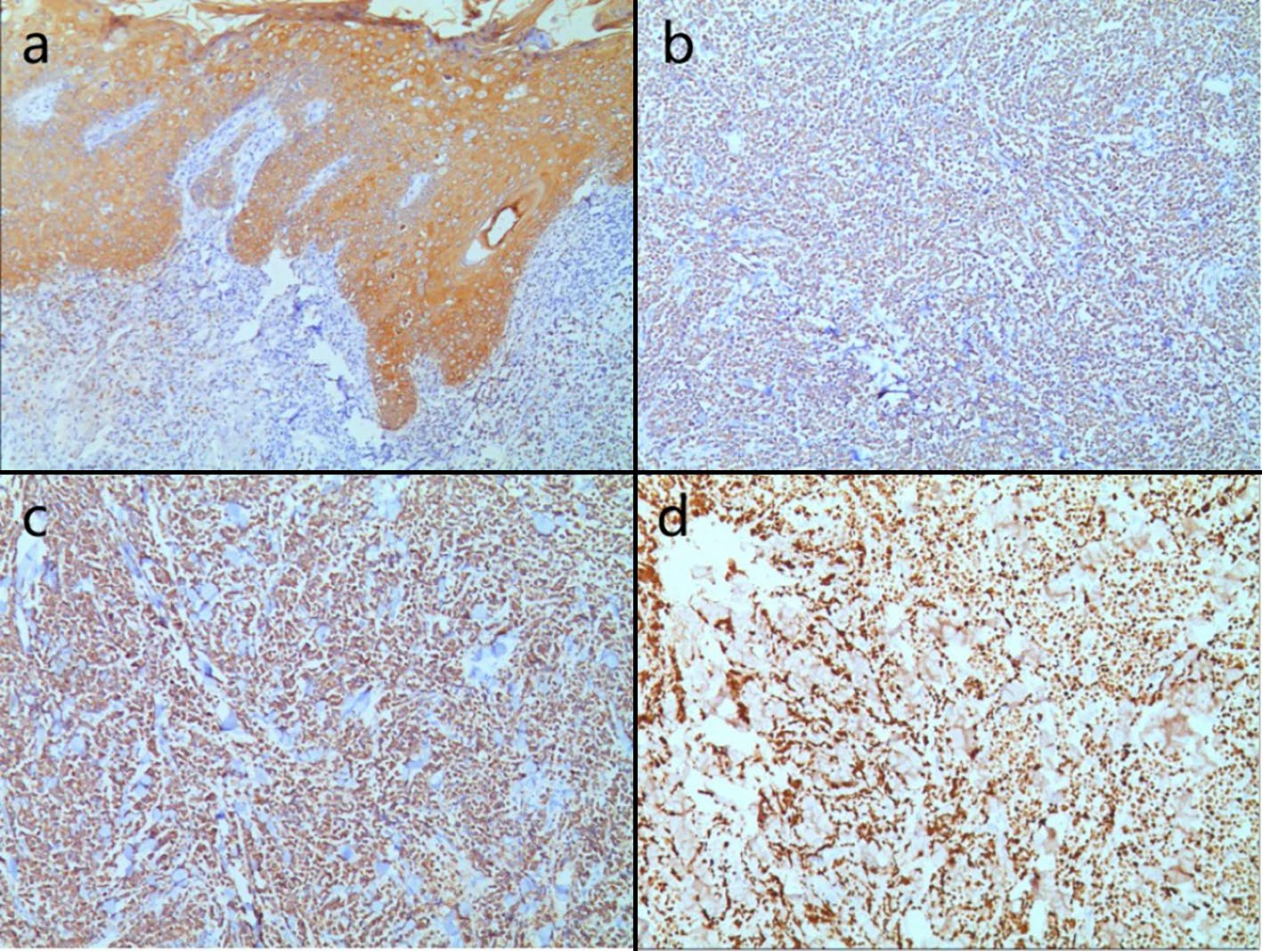
**a–c** Immunohistochemical staining showed that CK, CK20 and Syn were positive Magnification: ×10. **d** Immunohistochemical staining showed that Ki67 was positive in 90% of the cells Magnification: ×10

**Fig. 4:**
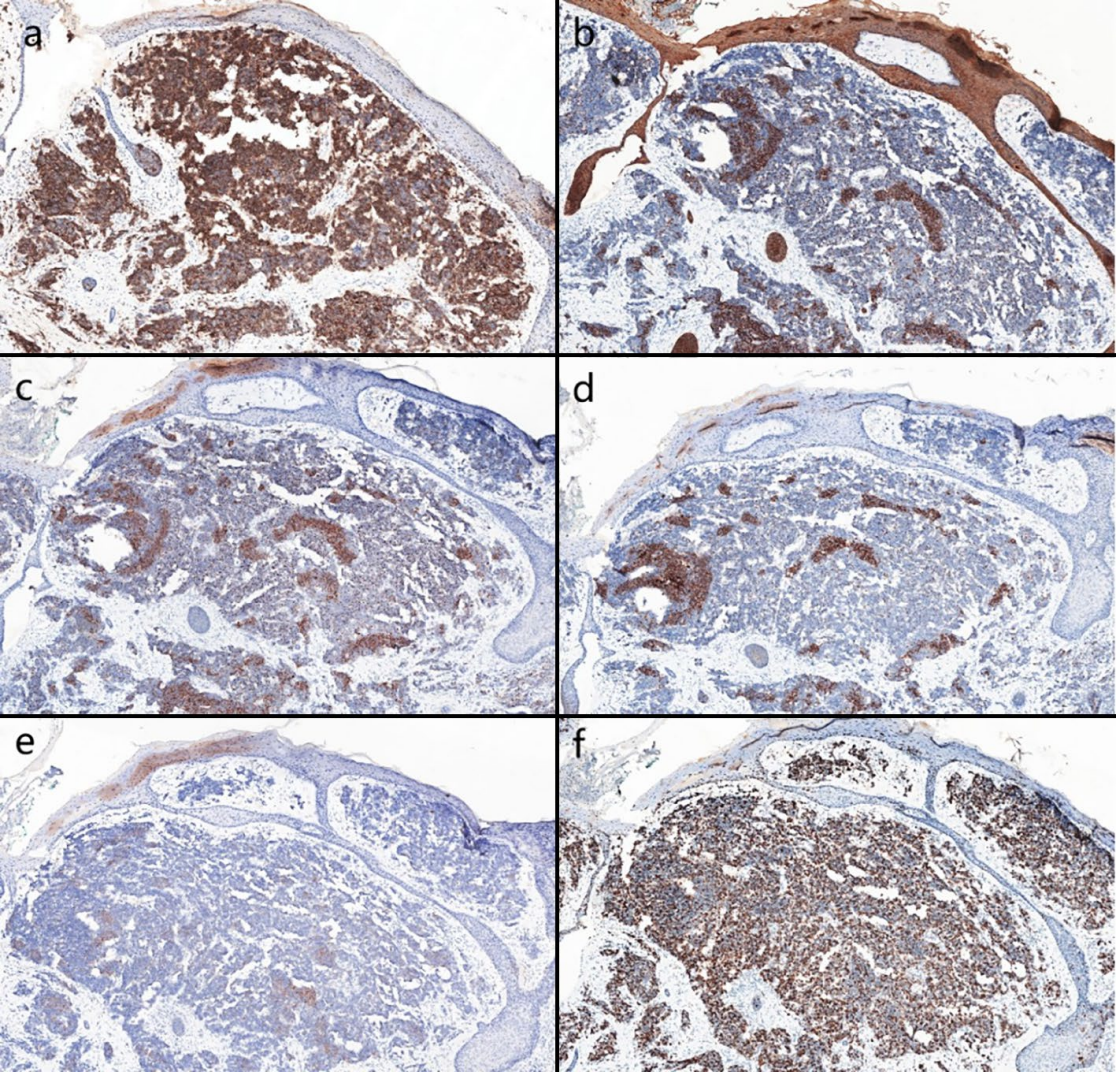
**a** Immunohistochemical staining showed CD56 positive Magnification: ×10. **b–e** Immunohistochemical staining showed that CK, CK20, CAM5.2 and CgA were focally positive Magnification: ×10. **f** Immunohistochemical staining showed that Ki67 was positive in 80% of the cells Magnification: ×10

Biopsies were performed respectively on the neoplasms in both cases. Both the histopathological examination of the two cases revealed gross hyperkeratosis with parakeratosis overlying a thickened dysplastic epidermis, with the atypical mitoses and multinucleated tumor giant cells. A small blue cell tumor extended deeply into the subcutaneous fat under the low-power magnification, and the pathognomonic tumor nuclei were large and pale staining and contain tiny nucleoli (Fig. 2a–f). Immunohistochemistry of case one showed positivity for CK, CK20, EMA, Synaptophysin (Syn) and Bcl-2 (Fig. 3a–d). Vimentin was positive in the stroma, while CD3, CD4, CD8 and CD20 showed scattered positivity. Ki67 was positive in 90% of tumor cells. LCA, CD68, CD30, TdT, CD56, Mum-1, TIA1, Granzyme B, EBER, Neuron-Specific Enolase (NSE), Chromogranin A (CgA) and CD79a were negative. In case two, immunohistochemistry showed positivity for CD56, focal positivity for CK, CK20, CAM5.2 and CgA, and negativity for Syn and CK7. Ki67 was positive in 80% of cells (Fig. 4a–f)

Both patients were diagnosed with MCC overlapping BD. They all underwent surgical excision extending 1 cm beyond the tumor margins. There was no recurrence during a follow-up period of 3 years in case one and about half a year in case two.

### Literature review

#### The demographic data

In the last 11 years (from 2013 to 2023), only 13 cases (including our two cases) of MCC overlapping BD have been described in the literature. The incidence was slightly higher in females than in males, with a male-to-female ratio of 1:1.6 (5 cases to 8 cases). The age ranged from 32 to 87 years (mean 72 years, median 73 years). Lesions mainly occurred at the age of more than 70 years (77%), and only one extremely rare case occurred at the age of 32 years. The overall duration of the disease varied from 2 months to 5 years. Some patients had a history of annual herbal pill consumption, exposure to ultraviolet radiation, and previous diagnoses of multiple myeloma, basal cell carcinoma, and BD (Choe et al. 2014; Miraflor et al. 2016) (Table 1).

**Table 1:** Characteristics of 13 cases of MCC overlapping BD of the last 11 years literature (2013–2023)

Authors & year of publication	Age/Sex	Involved site	Skin lesion	Onset	Medical history	Metastases	Treatment	Final outcome
Ishida et al. (2013)	86/M 87/F	In the chest In right cheek	A reddish tumor and focal erosion A nodular lesion	Unreported	Unreported	Yes, lymph node Unreported	SE SE	Unreported
Choe et al. (2014)	77/F	Right inguinal area	Dark brown plaque and reddish nodule on irregular brownish patch	About 2 years ago	Intake of herbal pill once or twice a year	Yes, lymph node, liver	SE, RT, and chemotherapy	Under treatment
Yamamoto (2014)	71/F	On the center of the left cheek	A dome-shaped reddish nodule	Unreported	No	No	SE	Unreported
Tono et al. (2015)	71/M	On the back	A red, pigmented plaque, with a dome-shaped elastic-soft nodule	6 months	Unreported	No	SE	Unreported
Miraflor et al. (2016)	71/M	On the left zygoma	A 0.3 cm pink, pearly papule with surrounding hypopigmentation	Not quite clear	MM of the back and basal cell carcinoma in the neck	No	SE	Follow up for 1 year without recurrence
McGowan et al. (2016)	73/F	On the right jawline	An ulcerated and violaceous 10-mm plaque	2 to 3 months	Unreported	No	SE	Unreported
Casari et al. (2018)	85/F	On the left cheek	Red and ulcerated nodule	Patch from 2 years ago, ulcerated nodule from 3 weeks ago	Ultraviolet radiation	No	SE	Unreported
Jeong et al. (2018)	82/F	On the right mandibular angle	An ovoid rubbery erythematous painless tumor mass	An abruptly enlarging reddish mass 2 months	Bowen’s disease	Unreported	Unreported	Unreported
Kiyohara et al. (2019)	65/M	On the left leg	A dome-shaped nodule, and the base of the nodule was contiguous with a dull brown plaque	Not quite clear	No	Yes, lymph node	SE, skin graft, avelumab	Follow up for 6 months without local recurrence or metastasis
Swain et al. (2022)	32/F	On the dorsum of the hand	Reddish ulcerated nodule	2 months	No	Yes, lymph node	SE, RT	Follow up for 7 years without recurrence
Our case (2023)	51/M 87/F	On the right waist On the right mandibular angle	Dark reddish nodule on erythematous scaly patch An ovoid dark erythematous tumor mass	Patch from 5 years ago, ulcerated nodule from 1 year ago About 1 year ago	No No	No No	SE SE	Follow up for 3 years without recurrence Follow up for 6 months without recurrence

Number of patients, age (years), distribution (face, trunk, upper or lower extremities, groin), clinical presentation, medical history, metastasis, therapy, follow-up, local recurrence,* F* female, *M* male, *SE* surgical excision, *RT* radiation therapy

#### Clinical manifestations

All the studies reported the location of the lesions. MCC overlapping BD were mostly located on the faces (*N* = 7/13, 53.8%), followed by the trunk (*N* = 3/13,23.1%), the upper extremity (*N* = 1/ 13,7.7%) , lower extremity (*N* = 1/ 13,7.7%) and groin (*N* = 1/ 13,7.7%). No patient had multiple lesions (Table 1).

Information regarding the initial clinical presentation was available for all patients. The lesions were most frequently described as asymptomatic, firm, dull red nodules on red or dark brown patches with frequent rapidly growing behavior, or as solitary nodules. None of the lesions described with accuracy were correctly diagnosed before biopsy and histological examination. The size of the tumor lesions was available for 11 lesions (84.6%). Tumor diameters ranged from 0.3 to 6.5 cm (mean: 2 cm, median: 1 cm). Rapid growth, either of new lesion or stable lesion from several months was the most frequent motivation for biopsy and diagnosis (Table 1).

Locoregional or distant metastases occurred in four patients (30.8%) (Swain et al. 2022; Choe et al. 2014; Ishida et al. 2013; Kiyohara et al. 2019). One patient showed lymph node and liver metastasis (Choe et al. 2014). After local excision of the cutaneous lesion and left inguinal lymph node dissection in one patient, several dermal and subcutaneous nodules developed successively on the left lower extremity (Kiyohara et al. 2019) (Table 1).

All 13cases had a histological diagnosis of MCC overlapping BD (Table 1). Eight patients were diagnosed by histopathology, followed by extensive local excision treatment. Of them, an 82-year-old female was diagnosed with MCC overlapping BD by histopathology but refused further evaluation and operative treatment (Jeong et al. 2018). Seven patients underwent direct extensive local excision treatment, followed by histopathological detection of the tissue post-surgery (Tono et al. 2015; Swain et al. 2022; Choe et al. 2014; Miraflor et al. 2016; Ishida et al. 2013; Kiyohara et al. 2019; Yamamoto 2014; McGowan et al. 2016; Casari et al. 2018). A 77-year-old woman was found to have lymph node and liver metastases after surgical treatment, followed by radiation and chemotherapy (Choe et al. 2014). A 65-year-old Japanese man experienced recurrent skin lesions after local surgery and lymph node clearance. After receiving avelumab treatment for 2 months, all lesions disappeared completely. Subsequent follow-ups over six months showed no recurrence (Kiyohara et al. 2019). A 32-year-old lady underwent surgery, lymph node clearance and received radiation therapy. This patient had an axillary dissection because of a palpable lymph node. Two lymph nodes out of 14 showed metastatic deposits, hence the female patient received radiotherapy after which she is well and completely free of disease now, 7 years after the initial diagnosis (Swain et al. 2022). A 71-year-old Caucasian male remained recurrence-free during the 1-year follow-up after surgical treatment (Miraflor et al. 2016). The follow-up status for the remaining seven patients has not been reported.

#### Histopathology

Histopathologically MCC primarily locates within the dermis and can invade subcutaneous tissues. At low magnification, it appeared as a typical small round blue-cell tumor, comprising three different histologic subtypes: trabecular type, intermediate type and small cell type. Among them, the intermediate type was the most common. The tumor consisted of nodules and diffuse sheets of basophilic tumor cells with vacuolated, pale-staining nuclei containing small nucleoli. The cytoplasm was indistinct with common nuclear folding. The trabecular type, the least common, was composed of slender, uniformly shaped cells, often with nuclear folding. The small cells type was characterized by infiltrates of deeply staining 'oat cell-like' cells with significant cell fragmentation.

The histopathological feature of MCC overlapping BD include abnormal keratin-forming cells of BD within the epidermis and small round blue-staining cells of MCC in the dermis. There has been a case reported where the MCC component, in association with BD, was confined to the epidermis, referred to as “intraepidermal MCC”(Miraflor et al. 2016).

The immunohistochemical characteristics of MCC overlapping BD indicate that MCC cells express neuroendocrine markers such as NSE, CK20, Neurofilament (NF), CgA, and Syn. Most MCCs do not express Thyroid Transcription Factor-1 (TTF-1). On the other hand, BD commonly exhibits expression of squamous cell markers like CK5/6, CK10 and CK14.

#### Dermoscopy

Dermoscopic examination was only reported in two patients (Casari et al. 2018). Dermoscopic examination showed the presence of clustered dotted vessels over a reddish structureless area that was suggestive for the diagnosis of BD. Addittional dermoscopical characteristic of the nodule included an atypical vascular pattern with tortuous vessels overlying a whitish background.

## Discussion

MCC is a primary cutaneous neuroendocrine carcinoma, predominantly diagnosed in fair-skinned elderly populations. Characterized by its aggressive nature, MCC is particularly notorious for its tendency towards local recurrences and distant metastases (Siqueira et al. 2023). Interestingly, MCC lesions may coexist with, or be found in close proximity to, a variety of other neoplasms, including actinic keratosis, BD, invasive SCC, BCC and sweat gland tumors (Kervarrec et al. 2022; Hobbs et al. 2020). Clinically, cases of MCC coexisting with BD are very rare. This comprehensive review of the literature spanning the past decade has revealed a notably rare occurrence, identifying only 11 cases of MCC overlapping BD. At the same time, we reported another two cases with MCC overlapping BD in the paper.

The annual incidence rate of MCC is approximately 0.24 cases per 100,000 individuals, showing a trend of exponential increase. This rising incidence can be attributed to a confluence of factors, including demographic shifts towards an aging population, heightened use of immunosuppressive agents, significant improvements in diagnostic technologies facilitating earlier and more accurate detection, and a general enhancement in clinical vigilance and awareness regarding this malignancy (Mistry et al. 2023; Mohsen et al. 2023). Our retrospective examination has disclosed that incidences of MCC overlapping BD present a gender distribution, with a male-to-female ratio of 1:1.6. Notably, a higher prevalence is observed in females, a finding potentially attributable to the limited scope of the sample size. The pathological manifestations of MCC predominantly arise in individuals aged over 70, though a sporadic occurrence in middle-aged adults has been noted (Swain et al. 2022). Owing to the absence of a conclusive diagnosis in MCC patients before undergoing histopathological examination, recent research has offered valuable insights into the clinical presentation of MCC, coalescing into the AEIOU mnemonic for ease of recall. “A” stands for asymptomatic lesions, often presenting without pain or tenderness. “E” denotes rapid expansion, with lesions demonstrating notable enlargement over a period of just three months. “I” represents immunosuppression, a key risk factor, encompassing conditions such as HIV infection, post-solid organ transplantation, or chronic lymphocytic leukemia. “O” refers to individuals over the age of 50, a demographic showing increased susceptibility. Finally, “U” highlights ultraviolet exposure in fair-skinned individuals as a significant risk factor. In the context of MCC overlapping BD, we propose the “AEIOUN” guideline, wherein “N” signifies the rapid emergence of nodules on the foundation of erythematous patches, expanding upon the initial AEIOU criteria.The presence of three or more of these features warrants a heightened clinical suspicion of MCC or MCC overlapping BD, guiding the clinician towards appropriate diagnostic and therapeutic interventions (Siqueira et al. 2023; Brusasco et al. 2022; Mistry et al. 2023).

The etiology of MCC is multifactorial. Current research posits that infection with MCPyV and genetic mutations triggered by ultraviolet (UV) radiation are primary contributors to the pathogenesis of MCC (Yang et al. 2022). Specifically, UV radiation plays a critical role in the development of MCPyV-negative MCC cases. Intriguingly, MCC cases that present concurrently with SCC or BD predominantly lack MCPyV. Among the 13 cases of MCC overlapping BD that were reviewed, only three patients underwent testing for MCPyV, and all 3 cases were negative for MCPyV. This observation suggests a distinct oncogenic mechanism in composite MCC, diverging from the pathways observed in solitary MCC (Kervarrec et al. 2022). The simultaneous manifestation of MCC overlapping BD might be attributed to a confluence of various factors, including immune regulation and the intricacies of the tumor microenvironment (Chattopadhyay et al. 2020). Genetic predisposition also appears to play a significant role in this context. Moreover, the pathogenesis of these conditions may be exacerbated by immunosuppression, immune deficiency or immune system dysregulation. The interplay and communication among cells within the tumor microenvironment, particularly through the release of cytokines, are believed to significantly influence the coexistence of MCC overlapping BD. Furthermore, lifestyle choices and environmental exposures, such as to chemicals, ultraviolet radiation, or toxins, are potential contributory elements in the concurrent development of these dermatological conditions (Casari et al. 2018).

MCC is unequivocally diagnosed through histopathological examination, recognized as the definitive gold standard. This is often supplemented by immunohistochemical profiling to accurately distinguish MCC from other poorly differentiated neoplasms. A significant majority of MCC cases demonstrate cytokeratin expression, with approximately 95% exhibiting perinuclear and/or cytoplasmic positivity for CK20 or CAM5.2. Additionally, these carcinomas frequently express neuroendocrine markers, most notably Syn, CgA, CD56 and NF. In contrast, TTF-1 and CDX-2 are typically negative in MCC (Khanna et al. 2020). Emerging studies suggest that around 60% of MCC cases express the protein p63, which is potentially correlating with a decreased overall survival rate and a lower disease-specific survival rate of patients. CK7 expression is generally absent in MCC, while there are noteworthy instances of CK7 positivity. A notable aspect in the immunohistochemical landscape of MCC is positive for MCV, observed in about 55% to 90% of cases. Interestingly, MCV-negative cases frequently demonstrated a lack of NF expression, and they distinctively exhibit markers of follicular stem cells along with a higher incidence of p53 positivity, which are predominant in CK20-negative MCCs. In patients with MCC overlapping BD, a distinctive dual pathology is often revealed through marker expression. The immunohistochemical signature of MCC is characterized by the concurrent expression of epithelial markers, including AE/1AE3, CAM5.2 and a broad spectrum of cytokeratins, alongside neuroendocrine markers such as neurofilaments and neuron-specific enolase. In contrast, BD typically demonstrates positive immunoreactivity for markers like CK7 and p16. Notably, an uncommon immunophenotype has been documented in a case merging MCC overlapping BD. In some cases, the MCC cells exhibited an absence of CK20 expression while maintaining positivity for CK7.

This diagnostic approach assumes critical importance in instances where MCC coexists with other tumors derived from epidermal origins. In conducting histological evaluations, it is imperative to scrutinize for the co-occurrence of other tumor types, such as SCC (observed in up to 15% of cases) or BCC. A critical aspect of the assessment is determining the extent of epithelial involvement by the tumor, whether the tumor cells are situated within the epidermis or affect the cutaneous adnexa. Furthermore, a detailed examination of the tumor's morphological attributes is essential, including discerning whether the tumor presents as infiltrative or manifests as well-defined nodular formations, alongside evaluating the dimensions (ranging from small to large) and the particular morphology of the cells, which can determine the appropriate treatment strategies and the prognosis of the tumors. The extent of invasion, growth patterns, and overall prognosis of MCC tumors exhibit significant interrelations (Gonzalez et al. 2022). Notably, patients exhibiting sentinel lymph node involvement typically will face a more challenging prognosis. Similarly, individuals suffering from active hematologic malignancies or under immunosuppression are also likely to experience adverse outcomes. Research on the differential prognosis or metastasis rates between MCC and MCC overlapping BD remains scarce. However, the co-occurrence of MCC overlapping BD may signal a broader spectrum of skin damage and an elevated risk of recurrence or metastasis.

The treatment of MCC overlapping BD adheres to the principles established for MCC treatment (Green et al. 2022; Harvey et al. 2022). In the management of MCC, surgical excision is often considered the primary modality of treatment. This involves employing techniques such as Mohs micrographic surgery or its modified forms, ensuring a margin of 1–2 cm extending to the fascia or periosteum (Uitentuis et al. 2022). For metastatic, post-surgical residual, or recurrent MCC, a combination of radiotherapy and chemotherapy serves as effective adjunctive treatment modalities. In recent advancements, immunotherapies targeting various anti-tumor immune mechanisms, particularly therapies focused on the PD-1 and PD-L1 pathways, have emerged as frontline treatments for metastatic MCC (Harms et al. 2018; Becker et al. 2018). Agents such as Avelumab, Pembrolizumab, and Nivolumab have been instrumental in significantly prolonging patient survival in these cases (Fojnica et al. 2023; Topalian et al. 2020).

## Conclusions

The coexistence of MCC overlapping BD represents an exceedingly rare condition, necessitating further research and accumulation of cases to better comprehend its clinical characteristics and determine the optimal therapeutic regimen. It is a lesion with nonspecific features and dermoscopy evaluation can be helpful for improving the clinical suspicion. We introduce the concept of “AEIOUN” as a pioneering approach for the early identification of clinically suspicious lesions indicative of MCC overlapping BD. The excision of doubtful nodular lesions is mandatory especially in the elderly, because MCC overlapping BD has not only a tendency to recur locally, but it can also metastasize. However, due to its rarity, there are no well-defined guidelines for the management. Complete surgical excision with clear margins stands as the optimal therapeutic choice, complemented by adjuvant radiotherapy, chemotherapy, immunotherapy, or their combination. Regular follow-ups are strongly recommended to monitor the condition. These two case reports and the review of the literature can provide better awareness and management of this rare tumor.

## Data Availability

All data generated or analyzed during this study are included in this published article.

## References

[CR1] Becker JC, Stang A, Hausen AZ, Fischer N, DeCaprio JA, Tothill RW et al (2018) Epidemiology, biology and therapy of Merkel cell carcinoma: conclusions from the EU project IMMOMEC. Cancer Immunol Immunother 67(3):341–351 29188306 10.1007/s00262-017-2099-3 PMC6015651

[CR2] Brusasco M, Macchi S, DEG F, Mora E, Zucchi A, Feliciani C et al (2022) AEIOU not only merkel cell carcinoma. Ital J Dermatol Venerol 157(5):453–454 36213971 10.23736/S2784-8671.22.07200-0

[CR3] Casari A, Argenziano G, Piana S, Lallas A, Moscarella E, Lombardi M et al (2018) Merkel cell carcinoma arising on a pre-existing bowen’s disease: is it just by chance? G Ital Dermatol Venereol 153(2):273–275 26222729 10.23736/S0392-0488.17.04987-2

[CR4] Chattopadhyay S, Hemminki A, Forsti A, Sundquist K, Sundquist J, Hemminki K (2020) Second primary cancers in patients with invasive and in situ squamous cell skin carcinoma, kaposi sarcoma, and merkel cell carcinoma: role for immune mechanisms? J Invest Dermatol 140(1):48–55 31288011 10.1016/j.jid.2019.04.031

[CR5] Choe Y, Kim Y, Park H, Yoon H, Cho S (2014) A case of merkel cell carcinoma concurrent with bowen’s disease. Korean J Dermatol 53(2):169–171

[CR6] Fojnica A, Ljuca K, Akhtar S, Gatalica Z, Vranic S (2023) An updated review of the biomarkers of response to immune checkpoint inhibitors in merkel cell carcinoma merkel cell carcinoma and immunotherapy. Cancers (Basel) 15(20):5084 37894451 10.3390/cancers15205084 PMC10605355

[CR7] Garcia-Carbonero R, Marquez-Rodas I, de la Cruz-Merino L, Martinez-Trufero J, Cabrera MA, Piulats JM et al (2019) Recent therapeutic advances and change in treatment paradigm of patients with merkel cell carcinoma. Oncologist 24(10):1375–1383 30962295 10.1634/theoncologist.2018-0718 PMC6795164

[CR8] Gonzalez MR, Bryce-Alberti M, Portmann-Baracco A, Castillo-Flores S, Pretell-Mazzini J (2022) Treatment and survival outcomes in metastatic merkel cell carcinoma: analysis of 2010 patients from the SEER database. Cancer Treat Res Commun 33:100665 36446191 10.1016/j.ctarc.2022.100665

[CR9] Green C, Isaksson Mettavainio M, Kjellman C, Ramqvist T, Dalianis T, Israelsson P et al (2022) Combined treatment with radiotherapy, chemotherapy and avelumab results in regression of metastatic merkel cell carcinoma and improvement of associated lambert-eaton myasthenic syndrome: a case report. Oncol Lett 24(5):393 36276497 10.3892/ol.2022.13513 PMC9533363

[CR10] Harms PW (2017) Update on merkel cell carcinoma. Clin Lab Med 37(3):485–501 28802497 10.1016/j.cll.2017.05.004

[CR11] Harms PW, Harms KL, Moore PS, DeCaprio JA, Nghiem P, Wong MKK et al (2018) The biology and treatment of Merkel cell carcinoma: current understanding and research priorities. Nat Rev Clin Oncol 15(12):763–776 30287935 10.1038/s41571-018-0103-2 PMC6319370

[CR12] Harvey JA, Mirza SA, Erwin PJ, Chan AW, Murad MH, Brewer JD (2022) Recurrence and mortality rates with different treatment approaches of merkel cell carcinoma: a systematic review and meta-analysis. Int J Dermatol 61(6):687–697 34227108 10.1111/ijd.15753

[CR13] Hobbs MM, Geers TE, Brown TS, Malone JC (2020) Triple collision tumor comprising merkel cell carcinoma with an unusual immunophenotype, squamous cell carcinoma in situ, and basal cell carcinoma. J Cutan Pathol 47(8):764–767 32243639 10.1111/cup.13698

[CR14] Ishida M, Okabe H (2013) Merkel cell carcinoma concurrent with bowen’s disease: two cases, one with an unusual immunophenotype. J Cutan Pathol 40(9):839–843 23672777 10.1111/cup.12176

[CR15] Jeong I, Kim T, Lee H (2018) Merkel cell carcinoma originating in a setting of pre-existing bowen’s disease. Korean J Dermatol 56(8):525–527

[CR16] Kervarrec T, Appenzeller S, Samimi M, Sarma B, Sarosi EM, Berthon P et al (2022) Merkel cell polyomavirus-negative merkel cell carcinoma originating from in situ squamous cell carcinoma: a keratinocytic tumor with neuroendocrine differentiation. J Invest Dermatol 142(3):516–527 34480892 10.1016/j.jid.2021.07.175

[CR17] Khanna U, North JP (2020) Large-cell variant of merkel cell carcinoma with clear-cell change. J Cutan Pathol 47(1):1–5 31840299 10.1111/cup.13583

[CR18] Kiyohara T, Shijimaya T, Miyamoto M, Nagano N, Nakamaru S, Makimura K et al (2019) In-transit recurrence of merkel cell carcinoma associated with bowen’s disease: the first reported case successfully treated by avelumab. J Dermatol 46(5):440–443 30809835 10.1111/1346-8138.14829

[CR19] McGowan MA, Helm MF, Tarbox MB (2016) Squamous cell carcinoma in situ overlying merkel cell carcinoma. J Cutan Med Surg 20(6):563–566 27207346 10.1177/1203475416649161

[CR20] Miraflor AP, LeBoit PE, Hirschman SA (2016) Intraepidermal merkel cell carcinoma with pagetoid bowen’s disease. J Cutan Pathol 43(11):921–926 27758029 10.1111/cup.12813

[CR21] Mistry K, Venables ZC (2023) A systematic review on merkel cell carcinoma epidemiology highlights the rising incidence, poor prognosis and data heterogeneity. Br J Dermatol. 10.1093/bjd/ljad434 37950910 10.1093/bjd/ljad434

[CR22] Mistry K, Levell NJ, Hollestein L, Wakkee M, Nijsten T, Knott CS et al (2023) Trends in incidence, treatment and survival of merkel cell carcinoma in England 2004–2018: a cohort study. Br J Dermatol 188(2):228–236 36763882 10.1093/bjd/ljac044

[CR23] Mohsen ST, Price EL, Chan AW, Hanna TP, Limacher JJ, Nessim C et al (2023) Incidence, mortality, and survival of merkel cell carcinoma: a systematic review of population based-studies. Br J Dermatol. 10.1093/bjd/ljad404 10.1093/bjd/ljad404 37874770

[CR24] Siqueira SOM, Campos-do-Carmo G, Dos Santos ALS, Martins C, de Melo AC (2023) Merkel cell carcinoma: epidemiology, clinical features, diagnosis and treatment of a rare disease. An Bras Dermatol 98(3):277–286 36870886 10.1016/j.abd.2022.09.003 PMC10173065

[CR25] Swain M, Yadav A, Pendharkar D, Patnaik S (2022) Concurrent merkel cell carcinoma and bowen’s disease in a young lady. Indian J Dermatol 67(6):761–763 36998867 10.4103/ijd.ijd_926_20 PMC10043676

[CR26] Tono H, Fujimura T, Iwama E, Kusakari Y, Furudate S, Kambayashi Y et al (2015) Merkel cell carcinoma concomitant with invasive bowen’s disease: immunohistochemical investigation of tumor-infiltrating leukocytes. Case Rep Dermatol 7(1):1–6 25759650 10.1159/000371732 PMC4327548

[CR27] Topalian SL, Bhatia S, Amin A, Kudchadkar RR, Sharfman WH, Lebbe C et al (2020) Neoadjuvant nivolumab for patients with resectable merkel cell carcinoma in the checkmate 358 trial. J Clin Oncol 38(22):2476–2487 32324435 10.1200/JCO.20.00201 PMC7392746

[CR28] Uitentuis SE, Bambach C, Elshot YS, Limpens J, van Akkooi ACJ, Bekkenk MW (2022) Merkel cell carcinoma, the impact of clinical excision margins and mohs micrographic surgery on recurrence and survival: a systematic review. Dermatol Surg 48(4):387–394 35165221 10.1097/DSS.0000000000003402

[CR29] Yamamoto T (2014) Epidermotropic pagetoid spread and squamous cell carcinoma in situ in the overlying epidermis of merkel cell carcinoma. Our Dermatol Online 5(2):210–211

[CR30] Yang A, Wijaya WA, Yang L, He Y, Cen Y, Chen J (2022) The impact of merkel cell polyomavirus positivity on prognosis of merkel cell carcinoma: a systematic review and meta-analysis. Front Oncol 12:1020805 36248985 10.3389/fonc.2022.1020805 PMC9562919

